# Lung Cancer Screening with Computer Aided Detection Chest Radiography: Design and Results of a Randomized, Controlled Trial

**DOI:** 10.1371/journal.pone.0059650

**Published:** 2013-03-20

**Authors:** Peter J. Mazzone, Nancy Obuchowski, Michael Phillips, Barbara Risius, Bana Bazerbashi, Moulay Meziane

**Affiliations:** 1 Respiratory Institute, Cleveland Clinic, Cleveland, Ohio, United States of America; 2 Quantitative Health Sciences, Cleveland Clinic, Cleveland, Ohio, United States of America; 3 Imaging Institute, Cleveland Clinic, Cleveland, Ohio, United States of America; 4 Imaging Institute, Cleveland Clinic, Abu Dhabi, United Arab Emirates; The University of Texas M. D. Anderson Cancer Center, United States of America

## Abstract

**Introduction:**

The sensitivity of CT based lung cancer screening for the detection of early lung cancer is balanced by the high number of benign lung nodules identified, the unknown consequences of radiation from the test, and the potential costs of a CT based screening program. CAD chest radiography may improve the sensitivity of standard chest radiography while minimizing the risks of CT based screening.

**Methods:**

Study subjects were age 40–75 years with 10+ pack-years of smoking and/or an additional risk for developing lung cancer. Subjects were randomized to receive a PA view chest radiograph or placebo control (went through the process of being imaged but were not imaged). Images were reviewed first without then with the assistance of CAD. Actionable nodules were reported and additional evaluation was tracked. The primary outcome was the rate of developing symptomatic advanced stage lung cancer.

**Results:**

1,424 subjects were enrolled. 710 received a CAD chest radiograph, 29 of whom were found to have an actionable lung nodule on prevalence screening. Of the 15 subjects who had a chest CT performed for additional evaluation, a lung nodule was confirmed in 4, 2 of which represented lung cancer. Both of the cancers were seen by the radiologist unaided and were identified by the CAD chest radiograph. The cumulative incidence of symptomatic advanced lung cancer was 0.42 cases per 100 person-years in the control arm; there were no events in the screening arm.

**Conclusions:**

Further evaluation is necessary to determine if CAD chest radiography has a role as a lung cancer screening tool.

ClinicalTrials.gov identifier NCT01663155

## Introduction

Lung cancer is most curable when detected at an early stage. Unfortunately, the majority of individuals with lung cancer present at an advanced stage, when the prognosis is very poor. Thus, fewer than 1 in 6 lung cancer patients will be living 5 years after their diagnosis [1]. Such a dismal prognosis remains despite major efforts in all fronts of the fight against lung cancer (prevention, detection and treatment). For these reasons there has been a tremendous investment in the study of imaging based lung cancer screening.

The major goal of any screening program is a reduction in the number of disease specific deaths in the screened population. Until recently, no lung cancer screening study was able to claim this outcome had been met. Controlled trials of chest x-ray based imaging showed improved survival but not reduced mortality [2]. CT cohort studies showed promising survival data but their design did not allow a mortality outcome to be assessed [3], [4]. These studies also highlighted issues with CT based screening, including the high number of benign lung nodules found requiring additional testing, the potential long-term risk of radiation from CT imaging, and the uncertain cost-effectiveness of a CT based screening program [3]–[5].

In 2008, prior to the announcement of any results of controlled trials of CT based screening, we developed a lung cancer screening trial in hopes of addressing some of the limitations of both standard chest x-ray and CT imaging, which could complement the ongoing controlled trials of CT screening. As an alternative to standard chest x-ray and CT screening, the use of a chest x-ray system with an improved ability to detect lung cancer could have some advantages. Chest x-rays are readily available, less costly, identify fewer false positives, and subject patients to less radiation. Computer aided detection (CAD) of lung nodules on chest x-rays has the potential to improve the sensitivity of standard chest x-rays to detect early lung cancer. Here we describe the study protocol and results of screening using a CAD chest x-ray system. The primary objective of this study was to determine if lung cancer screening with a CAD chest x-ray system would lead to a reduction in symptomatic advanced stage lung cancer. To our knowledge this was the first controlled trial of chest x-ray screening in which there is a placebo control group, and the first chest x-ray screening trial that used CAD to improve the ability to detect subtle cancers.

## Methods

### Ethics Statement

The study was approved by the Institutional Review Board of the Cleveland Clinic. All study participants signed an informed consent document.

### Trial Design

This was a randomized, placebo-controlled trial of lung cancer screening with a CAD chest x-ray system in an at-risk population. This was a single center study with 4 locations where imaging occurred through the Cleveland Clinic Health System. The lower end of the age criteria was reduced from 50 to 40 early in the trial due to slow recruitment. The trial was terminated early, upon report of the NLST trial results, because of both slow recruitment and a determination that an evolution of the trial to one comparing CAD chest x-ray to low-dose CT had become more relevant.

### Study Objectives

The primary objective of the study was to determine whether lung cancer screening with CAD chest x-rays reduces the incidence of symptomatic advanced lung cancer compared to no screening in a high-risk population. The presence of advanced symptomatic lung cancer was based on meeting all of the following criteria: The patient has been diagnosed with non-small cell carcinoma, stage II or higher, or small cell carcinoma, any stage; The patient has experienced a symptom that led them to seek contact with a physician since the time of the last study visit, or at the time of their next study visit the patient had developed a new symptom, or a change in a chronic symptom within the prior 6 weeks, that did not yet lead to a doctor’s visit; The symptom(s) are felt to be related to the diagnosis of lung cancer as adjudicated by an Outcomes Review Committee.

### Inclusion Criteria

1. Ages 40–75 years and at least one of the following criteria:
  - A current or ex-smoker with at least a 10 pack year history.
  - A first degree family member (parent, sibling, or child) with a history of lung cancer.
  - A clinical diagnosis of chronic obstructive pulmonary disease (COPD).
2. Able to return for annual follow-up screening.
3. Willing to sign a medical release form.

### Exclusion Criteria

1. Within the 6 weeks preceding enrollment, subject has had:
  - A new cough or chronic cough that has worsened.
  - New shortness of breath, or any worsening of shortness of breath.
  - A cough producing blood.
  - Constant chest pain.
  - Respiratory infection.
  - Unintentional and unexplained weight loss greater than 5% of total body weight.
2. Current health condition requires the use of supplemental oxygen.
3. Subject has a medical condition that would prevent the subject from undergoing treatment for lung cancer.
4. Subject has been diagnosed with a malignancy within the last 5 years, excluding non-melanoma skin cancer, carcinoma in situ of the cervix and localized prostate cancer.
5. Subject has received a chest x-ray or chest CT within the last 6 months.
6. Subject is participating in another cancer screening trial, an investigational drug or device study, or a cancer prevention study.
7. Subject has had a pneumonectomy.
8. Subject has had a lobectomy or segmentectomy within the last 5 years.

### Study Subject Randomization

Study subjects were randomized with equal allocation to either screening with a CAD chest x-ray system or a placebo screening procedure (standing for a chest x-ray but none taken). A 4-variable stratified randomization design was applied based on the following stratification variables: site of enrollment (Cleveland Clinic main campus, east, west, or south satellite clinics), age (<65 versus ≥65 years of age), gender, and symptoms status (no reported chronic cough or shortness of breath versus reported chronic cough or shortness of breath). There were 8 strata within each enrollment site: 2 ages×2 genders×2 symptom statuses×4 locations (total of 32 strata). Within each stratum we used a randomized block of variable size for that stratum. After enrollment study subject information was entered into a database leading to an automatic central assignment to chest x-ray or placebo arms.

### The Chest X-ray Procedure

Patients randomized to the screening arm received a standard frontal PA view chest x-ray. The technical factors were 125 kVp, 1.60 mAs, 500 ms, left and right AEC sensors, 72″ SID. The radiation dose to the patient is 20 millirems for the frontal view. The image was then sent electronically to be archived and to a dedicated PACS reading station for interpretation. The same image was sent simultaneously to a CAD server where the image was processed and regions of interest were marked. The CAD-processed x-ray was available for review within seconds. The CAD version used was OnGuard 5.0 (Riverain Medical).

Patients randomized to the control group were introduced to a CXR room and placed against the x-ray unit. The aiming light was positioned properly and a clicking sound occurred when the radiology technician initiated the placebo x-ray, however no exposure was triggered and no image was obtained.

### Chest X-ray Interpretation

Chest x-rays were interpreted by chest radiology specialists. The radiologist first read the case without and then with the assistance of CAD. The report by the radiologist included the reader findings and recommendations for follow-up. If the radiologist did not find an actionable finding and did not recommend follow-up for the subject, then the images were read by a second chest radiologist. This second reading was blinded to the first interpretation. The interpretations were registered electronically into a secure database. Subjects who underwent a chest x-ray and were found to have an actionable finding on the chest x-ray by one of the radiologists were mailed a report of the findings via certified mail. An actionable finding was defined as any finding the radiologist felt would require additional evaluation or follow-up. If the chest radiologist felt a finding required only chest x-ray follow-up at the 1 year mark a letter was not sent.

Subjects with actionable findings who were sent a letter were phoned after written notification to answer any questions the subject had, and to encourage a follow-up visit with a primary care physician or pulmonologist. The recommendations of the radiologist were not mandated to the subject’s physician; rather, subjects and their physicians together determined the plan for follow-up testing and treatment, as deemed appropriate.

### Annual Screening, Follow-up, and Data Collection

Study subjects were to receive annual screening procedures in their assigned group for a total of 3 incident screens. The procedure for these annual screens was as described above for the initial screen. Follow-up with data collection was to continue for at least 5 years. Data was to be collected throughout the study via the completion of study forms and/or direct phone calls at 6 month intervals.

The baseline form collected data on age, race, gender, education level, work status, income range, previous and existing medical conditions, family history of medical conditions, and occupational exposures. Every 6 months all subjects completed a follow-up form and a medical utilization form to capture data on physician visits in the previous 6 months. If any study subject reported that they have been diagnosed with lung cancer, their medical records were obtained.

A radiology form was to be completed by the chest radiologist at baseline and years 1, 2 and 3. Information collected included the number of actionable nodules, their location, size, contour, shape, non-calcified density, and notable findings.

### Statistical Analysis

A reduction in the development of symptomatic advanced stage cancer was the primary end point. Thus we considered lung cancer prevalence and incidence in the study population, as well as an estimation of the ability to detect lung nodules potentially representing early stage lung cancer with the CAD chest x-ray system, in our sample size calculation (Table 1). The prevalence of screened detected cancers in cohorts of smokers with 20 or more pack-years is 1.06–2.03% [3]; this range likely underestimates the true prevalence rate because the sensitivity of screening is <1. Similarly, the reported annual incidence of lung cancer is 0.5–1.4% [3]. For our power calculation, we used a baseline prevalence rate of 2% and an annual incidence rate of 0.5%. We assumed a non-lung cancer annual mortality rate of 1.4% [6]. With two fellowship-trained radiologists interpreting the x-rays with CAD in sequence, assuming a sensitivity of 0.85 for each and independence, the estimated sensitivity is (0.85)+(0.15×0.85) = 0.978. We assumed a very low rate, 1%, of subjects would be misdiagnosed after follow-up imaging and thus would undergo needless invasive testing. We assumed a 30-day mortality rate after lung resection of 2%, which was higher than our institution’s rate. With 4000 subjects per study arm we calculated that we would have 90% power to detect a reduction in the primary endpoint of 50% or higher.

**Table 1:** The assumptions made in calculating the required sample size for this study.

Prevalence of lung cancer at baseline screening	0.02
Annual incidence of lung cancer	0.005
Annual lost to follow-up rate	10%
Duration of Recruitment	2 years
# of annual incident screens	3
non-lung cancer annual mortality rate	0.014
sensitivity of x-ray with CAD read in series by 2 fellowship-trained radiologists	0.978
% of patients without lung cancer who are falsely diagnosed on x-ray and follow-up testing and undergo invasive testing	1%
% of screened patients who are compliant with screening	90%
30-day mortality rate following lung surgery	0.02
% reduction in development of symptoms due to early detection	50%

With 4000 subjects per study arm, we expected to have 90% power at 5 years to detect a 50% reduction in symptomatic lung cancer [14].

The observed cumulative incidence of symptomatic advanced lung cancer, measured in events per person-years observed, was to be compared between the two study arms. The analysis was to be carried out from an intent-to-treat perspective. A stratified analysis using age of 65 as a cutoff and the presence versus absence of baseline chronic symptoms was to be used. A similar analysis was to be performed for lung cancer specific and all-cause mortality.

A Cox proportional hazards model was to be fit to compare the two study arms on the time from randomization until symptomatic disease. Covariates were to be included in the model, such as age, gender, race, smoking history, chronic symptoms at presentation, as well as site of enrollment. A significance level of 0.05 was to be applied.

Two interim analyses, at the end of year 3 and the end of year 4, were planned. The final analysis was planned to occur at the end of the fifth year. The “use function” of Lan and DeMets [7] was to be used to generate group sequential boundaries analogous to the boundaries of O’Brien and Fleming [8]. The Lan-DeMets method allows us to perform the interim analyses at specified calendar time points based on the proportion of events (of the total number of events accrued by the end of five years) that have occurred at the time of the interim look. The overall significance level was to be 0.05.

For the current analyses, continuous variables were compared between groups using two-sample t-tests, ordinal variables were compared using a Wilcoxon two-sample test, and categorical variables were compared using chi-square or exact tests. The sensitivity of the CAD chest x-ray to identify a region of interest at the site of an actionable nodule and at the site of a subsequently confirmed lung cancer is reported.

## Results

**Figure 1:**
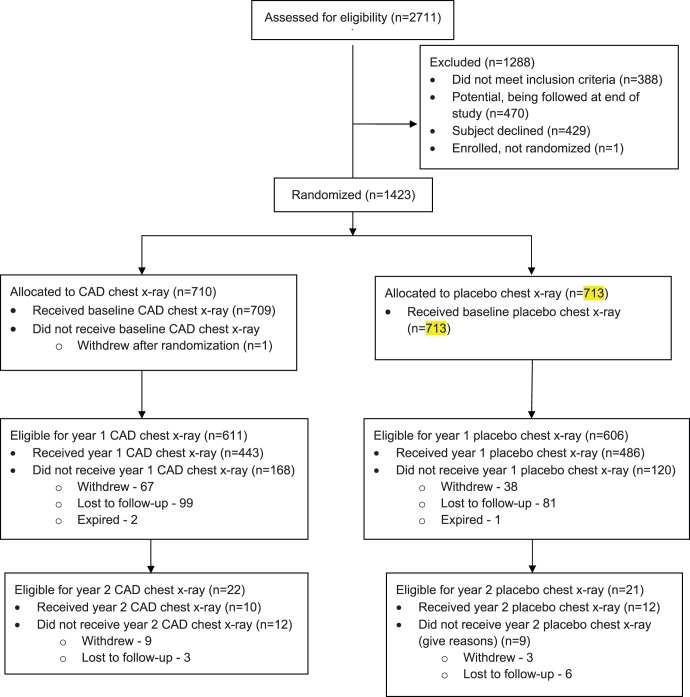
Flow of study subjects through the trial.

From September 2008 through April 2011 1,423 subjects were enrolled and completed their baseline screen. Study participant characteristics are described in Table 2. Study subjects had been enrolled in the study from 1–31 months. 80.8% of subjects have follow-up data at 6 months, 66.5% at 12 months, 38.5% at 18 months, 1.8% at two years or beyond for a total of 1331.5 subject follow-up years. The flow of patients through the trial, up to the point the study was terminated, is outlined in Figure 1. The trial was terminated early, upon report of the NLST trial results, because of both slow recruitment and a determination that an evolution of the trial to one comparing CAD chest x-ray to low-dose CT had become more relevant.

**Table 2:** Characteristics of study subjects.

	Control group # (%)	Screened group # (%)	p-value
Number of participants	713 (100)	710 (100)	
Age – Mean (range)	60.2 (41.5, 77.7)	59.9 (42.5, 77.8)	0.45
Females	387 (54.3)	385 (54.2)	0.96
Smoking history			0.85
Active	361 (50.6)	366 (51.5)	
Never	11 (1.5)	13 (1.8)	
Former	341 (47.8)	332 (46.7)	
Pack-Years:			0.17
<20	118 (16.8)	125 (17.9)	
20–40	323 (46.0)	276 (39.5)	
40–60	172 (24.5)	193 (27.7)	
60–80	62 (8.8)	69 (9.9)	
>80	27 (3.8)	35 (5.0)	
Diabetes	58 (8.1)	61 (8.6)	0.76
Hypertension	238 (33.4)	217 (30.5)	0.25
CAD	139 (19.5)	144 (20.3)	0.72
CHF	4 (0.6)	14 (2.0)	0.017
CVA	12 (1.7)	20 (2.8)	0.15
COPD	95 (13.3)	122 (17.2)	0.04
Pulmonary fibrosis	1 (0.1)	3 (0.4)	0.32
Asthma	65 (9.1)	64 (9.0)	0.94
Kidney disease	9 (1.3)	9 (1.3)	>0.99
Fam hx lung cancer	182 (25.5)	183 (25.7)	0.93
Cough/shortness of breath (chronic)	251 (35.2)	252 (35.4)	0.92

Table 3 summarizes the prevalence screening findings in the screened arm (710 subjects). A total of 29 actionable nodules were noted in 29 patients at baseline. In 11 of these, the radiologist felt that 1 year f/u was indicated and thus a letter was not produced. In 22 of 29 cases (75.9%) the nodule was first seen unaided (11 by reader one and 11 by reader two). The remaining 7 were seen first with CAD (4 by reader one and 3 by reader two). CAD identified 19 of the 29 actionable nodules (65.5%). Table 4 summarizes the imaging characteristics of the actionable nodules. Those identified first by CAD were less likely to be solid (p = 0.038) and tended to be smaller (NS). The evaluation of these actionable nodules included a chest CT scan in 15 of the 18 patients who received a letter. The actionable nodule was confirmed to be present in 4 of these 15 patients. All 4 had been identified by reader 1 unaided. Two of these 4 were also identified by CAD. CT imaging identified an additional nodule requiring follow-up in 4 subjects. Two of the actionable nodules went on to be diagnosed as lung cancer. Both were identified unaided by reader 1 and had also been identified by CAD. In total, the evaluation of these 18 nodules included 7 chest x-rays, 28 chest CT scans, 3 PET scans, 3 non-surgical biopsies, and 2 surgical resections. An additional 90 subjects (12.6%) were labeled as having a granuloma based on the chest x-ray appearance of the nodule.

**Table 3:** Baseline findings from the CXRs of the screened group.

Finding	No. of Subjects (% of screen arm)
Actionable Nodule	29 (4.1)
Emphysema/COPD	112 (15.7)
Granuloma	90 (12.6)
Fibrosis	123 (17.3)
Pleural Abnormality	67 (9.4)
Calcified Lymph Nodes	11 (1.5)
Lymphadenopathy	2 (0.3)
Enlarged Heart	19 (2.7)
Rib fracture- healed	31 (4.4)
Interstitial Lung Disease	4 (0.6)
Aortic calcification	61 (8.6)
Aortic dilation	6 (0.8)
Hernia/diaphragm eventration	17 (2.4)
Enlarged Thyroid	1 (0.14)
Scoliosis	32 (4.5)
Non-Nodule Actionable Findings	6 (0.8)

**Table 4:** Characteristics of actionable nodules identified on the baseline screen.

		Detected Unaided (N = 22)	Detected with CAD (N = 7)	p-value
Size:				0.240
<5 mm	3	2 (9.1%)	1 (14.3%)	
5–10 mm	17	12 (54.6%)	5 (71.4%)	
11–15 mm	2	1 (4.6%)	1 (14.3%)	
16–20 mm	3	3 (13.6%)	0 (0%)	
>20 mm	4	4 (18.2%)	0 (0%)	
Density:				1.0
High	0	0 (0%)	0 (0%)	
Moderate	9	7 (31.8%)	2 (28.6%)	
Low	20	15 (68.2%)	5 (71.4%)	
Appearance:				0.038
Solid	22	19 (86.4%)	3 (42.9%)	
Infiltrating	7	3 (13.6%)	4 (57.1%)	
Contour:				0.47
Irregular	10	6 (27.3%)	4 (57.1%)	
Spiculated	1	1 (4.6%)	0 (0%)	
Smooth	15	13 (59.1%)	2 (28.6%)	
Lobulated	3	2 (9.1%)	1 (14.3%)	
Shape:				0.25
Round	14	12 (54.6%)	2 (28.6%)	
Oval	9	7 (31.8%)	2 (28.6%)	
Triangular	1	0 (0%)	1 (14.3%)	
Irregular	5	3 (13.6%)	2 (28.6%)	

In the first incidence round of screening (performed on 929 of 1217 eligible subjects), an additional 6 actionable nodules were identified. All 6 were identified unaided, 2 by reader 1 and 4 by reader 2. CAD identified 3 of the 6. 4 of the 6 were 5–10 mm in diameter, 1 11–15 mm, and the final >30 mm. 2 had irregular borders and 4 smooth. 4 were round, one oval and one irregular. Two of the nodules were felt to require a one year follow-up chest x-ray and thus did not trigger a letter. One did not receive additional imaging, another resolved on follow-up chest x-ray, one was not seen on chest CT, and the other appeared to be an area of focal fibrosis on chest CT.

As of April, 2011 4 subjects in the control arm were found to have lung cancer (2 squamous cell –1 stage 1A, 1 stage IIB, 1 stage IV adenocarcinoma, and 1 extensive stage small cell carcinoma), as were 2 subjects in the screening arm (1 stage IA adenocarcinoma, 1 stage IB squamous cell carcinoma). Upon review by the Outcomes Review Committee, there were three symptomatic advanced lung cancer events; all occurred in the control arm. One subject had an acute episode of back pain several days after randomization, one patient reported symptoms initially attributed to emphysema, COPD, and chronic bronchitis at the time of randomization then sought medical treatment 6 weeks after randomization, and one patient developed symptoms 7 months after randomization. The cumulative incidence of symptomatic advanced lung cancer was 0.42 cases per 100 person-years in the control arm; there were no events in the screening arm.

## Discussion

Lung cancer is an ideal disease for a screening program. It is of great public health importance, is detectable in a preclinical phase, treatment is available for early stage disease, and treatment is more effective when the disease is found at an early stage. Controlled trials of chest x-ray screening have failed to show a reduction in lung cancer specific mortality [2]. It is possible that the earliest stage lung cancers are missed on chest x-ray imaging, suggesting a more sensitive screening test could be more successful. This was confirmed by findings from the National Lung Screening Trial (NLST), demonstrating a 20% reduction in lung cancer specific mortality in those who received reduced dose CT screening [9]. Problems with CT screening include a high number of false positives (small indeterminate lung nodules) which require close surveillance and potentially invasive testing to evaluate, difficulty in determining risk from the radiation received during the test, and the relatively high cost of the test and evaluation of its results [3]–[5]. The study reported here was designed in an attempt to address some of these concerns. Chest x-ray CAD systems may improve upon the sensitivity of standard chest x-rays for the detection of early stage lung cancer, yet are unlikely to identify the very small benign lung nodules found on chest CT imaging. Chest x-ray is less costly and delivers a much lower dose of radiation than chest CT scans. In this manuscript we have described the study design of the first ever randomized, placebo controlled trial of lung cancer screening using a CAD chest x-ray system. The primary endpoint of the study was to be a reduction in symptomatic advanced stage lung cancer in the screened group.

The goal of using a CAD system is to maximize the sensitivity of the readers’ detection of small lung nodules. The challenge with all CAD systems is realizing improvement in sensitivity while minimizing the number of false positives. Many reports of CAD chest CT systems are available in the literature. Systems reported have shown accuracy in identifying lung nodules equal to that of an experienced radiologist. The combination of the CAD system and the radiologist is more accurate than either alone [10], [11]. There are few CAD systems for chest x-rays. The system used in this study has improved over time. In a study of multiple versions of the system, using CT identified small lung nodules as the gold standard, the systems’ sensitivity improved from 44% with an early version to 64% with a later version. At the same time the number of false positives fell from 3.9 per image to 2.0 per image [12]. When used in a retrospective study of multiple reader types (expert readers, general radiologists, and pulmonologists) the readers’ sensitivities could have improved by up to 21% if all true positive CAD findings were accepted. In the expert reader group the number of false positives did not increase by using the CAD system [13]. In the current report 7 of the 35 actionable nodules found in the screened group were identified first by the CAD system. Approximately half of the actionable nodules were identified by the second reader after the first reader had determined an actionable nodule did not exist. Both of the lung cancers were seen by the first expert radiologist and the CAD system. Of the 15 actionable nodules identified in the prevalence round that went on to CT imaging, 11 of these proved to be false positives. Seven of these 15 were identified by CAD as well, 5 of which were false positives. The other 2 were cancers. The number of lung cancers identified was too small to make any judgments about the intended primary endpoint. This data suggests that advances in the CAD technology, beyond the version used, are required before an impact of the technology could be expected, and that these advances could be of significant benefit to chest x-ray interpretation. The main limitation of the study was the slower than expected overall recruitment, leaving the study without enough power to assess the primary objective at the time the investigators decided to terminate and evolve the study.

With the announcement of the NLST results, we were faced with a decision on how to proceed with our screening trial. It was apparent that recruiting the number of subjects we required to adequately assess our primary outcome was difficult at baseline, and that it would become much more difficult to enroll subjects to a placebo controlled trial of CAD chest x-ray screening given the positive findings of the NLST. It was also apparent that all other screening modalities would need to be compared to the benefit shown by chest CT screening, and that this would not be possible with the resources available to complete our trial. As such, we decided to allow our trial to evolve in order to help answer some of the remaining questions. Most importantly, we will aim to determine if advances in CAD chest-ray imaging, and non-imaging biomarkers can evolve lung cancer screening through better lung cancer risk prediction, early identification, and characterization. For these purposes, we changed the study protocol to a direct cross-sectional comparison of chest x-ray CAD to reduced dose chest CT, and developed a biorepository. The digital chest x-ray images will be able to be stored allowing advances in CAD and other technologies to be applied and compared to the CT images over time. It is our hope that by the end of the study period clinical lung cancer screening programs will be developed and accepted, allowing this group to be followed for longer periods of time.

### Other Information

The protocol for this trial and supporting CONSORT checklist are available as supporting information; see Checklist S1 and Protocol S1. The trial was funded by the Ohio Department of Development, TECH 06-55. The funding source did not have an influence on the design of the trial, data analysis, or decision to publish.

## Supporting Information

Figure S1 (TIF)

Checklist S1 **CONSORT checklist.**
(DOC)

Protocol S1 **Study protocol.**
(DOC)
