# Supplementary material for: Lung Cancer Screening with Computer Aided Detection Chest Radiography: Design and Results of a Randomized, Controlled Trial
Source: PLoS One. 2013 Mar 20;8(3):e59650. doi: 10.1371/journal.pone.0059650 (PMC3603858; doi:10.1371/journal.pone.0059650)
Supplement: Protocol S1 — Study protocol. (DOC) [file pone.0059650.s003.doc]

STUDY TITLE: A Blinded, Randomized, Controlled Clinical Trial to Determine the Effect of Chest X-Ray Screening and Interpretation using Computer Aided Detection (CAD) on Advanced-Stage Lung Cancer Rate

VERSION Version 6.0 – November 5, 2009

PRINCIPAL Moulay Meziane MD

INVESTIGATORS: Radiology

Cleveland Clinic

Peter J. Mazzone MD

Pulmonary, Allergy & Critical Care Medicine Cleveland Clinic

CO- INVESTIGATORS: Omar Lababede MD

Radiology

Cleveland Clinic

Nathan Pennell, M.D., Ph.D

Hematology and Medical Oncology

Cleveland Clinic

Sudish Murthy MD

Cardiothoracic Surgery

Cleveland Clinic

STATISTICIAN: Nancy Obuchowski PhD

Cleveland Clinic

SPONSOR: Investigator Initiated

State of Ohio - Department of Development

**TABLE OF CONTENTS**

1.0 Abstract 3

2.0 Background and Rationale 3

3.0 Objectives 4

4.0 Selection of Patients (Inclusion and Exclusion Criteria) 5

5.0 Registration Procedure 6

6.0 Study Description and Randomization 6

7.0 Technical Description of Chest X-Ray 7

1. Interpretation of Chest X-Ray and CAD 7
2. Annual Screenings 8

10.0 Subject Follow-Up 9

11.0 Records Keeping 9

12.0 Endpoint Assessment 13

13.0 Statistical Analysis Plan 14

14.0 Sample Size Considerations 16

15.0 Cost-Effectiveness 18

16.0 Adverse Events 20

17.0 Data Safety and Monitoring Committee 21

18.0 Patient Consent and Peer Judgment 21

19.0 References 22

20.0 Appendices 24

1.0 **ABSTRACT**

A two-arm randomized clinical trial of 8,000 high-risk asymptomatic people is planned. Study subjects will be randomized with equal allocation to either A) screening with chest x-ray interpreted by chest radiologists using computer-aided detection (CAD), or B) placebo screening chest x-ray. Screening will occur at entry into the study and one annual screen thereafter. Expert-chest radiologists will first record their findings without CAD and then will record their findings with CAD. The primary aim of the study is to report and compare the frequency of advanced-stage lung cancers at the end of the 2 ½ year study between screened and placebo groups. Size and stage are important intermediary outcomes of screening. Logically, screening would need to detect lung nodules as smaller, earlier stage, treatable, lesions compared to lesions diagnosed after symptoms develop. A comparison of the frequency of advanced-stage cancers after screening, compared to no screening, allows us to assess the effect, if any, of screening on reducing the number of advanced-stage lung cancers.

2.0 **BACKGROUND AND RATIONALE**

Lung cancer is a major public health problem. In the United States approximately twenty nine percent of cancer deaths are secondary to lung cancer ^1^. There is an estimated 160,000 deaths from lung cancer in the United States each year and over 2 million worldwide. Most of these deaths could be avoided if people did not smoke tobacco related products. Unfortunately, worldwide tobacco consumption has not been declining; suggesting lung cancer will remain an epidemic for years to come. The latest statistics show that Ohio incidence rate of tobacco-related lung cancer was 75.4/100,000 and lung cancer death rate was 64/100,000, both higher than the national estimate. Also, Ohio has the 4^th^ highest prevalence of cigarette smoking (27.7% or 3.2 million Ohioans). Based on those statistics, the economic cost to the state of Ohio is estimated at more than 5 billion per year.^2, 3^

A high proportion of individuals with lung cancer present at an advanced stage, leaving the overall prognosis very poor once it has been detected. Just over 1 in 8 lung cancer patients will be living 5 years after their diagnosis. Such a dismal prognosis remains despite major efforts in all fronts of the fight against lung cancer (prevention, detection and treatment). The development of an effective lung cancer screening program in which imaging could play a central role would be a major public health achievement.

The earliest efforts at radiographic screening were from the analysis of mass chest x-ray (CXR) screenings from the population of an individual city. This was followed by large controlled studies in the 1970’s that used CXR, sputum, or a combination of the two, as screening tools. Despite considerable ongoing debate about the design and analysis of these randomized studies, they have been interpreted as not showing that screening with plain CXR and/or sputum examination has a beneficial effect on mortality from lung cancer. ^4^

Given the disappointing overall results from CXR as a screening technique to date, more recent efforts have centered on the use of low-dose CT imaging as a screening tool. Multiple cohorts of at risk subjects screened with chest CT have been reported. Important highlights of these studies include the ability to find many early stage lung cancers, and lengthy survival of those diagnosed with an early stage lung cancer. Difficulties identified in these studies are an inability to comment on lung cancer specific mortality, a large number of benign nodules being identified (5-50% of participants on the initial chest x-ray), intense testing protocols required to follow the identified nodules to ensure they are not cancer, invasive procedures performed on some benign nodules, and questionable cost-effectiveness. ^5-13^

As it is still to be proven whether CT screening will reduce overall and disease-specific mortality, current guidelines do not recommend lung cancer screening for asymptomatic individuals at risk for lung cancer. Individual patients at risk for lung cancer are being advised of their risk and educated about the current state of early detection. If testing is to occur, it should be in a trial setting in which multidisciplinary specialty groups exist. ^14, 15^ Two large randomized controlled trials of CT screening are ongoing. These should answer many of the remaining questions about screening for lung cancer with chest CT imaging.

An alternative approach to CT screening would be the use of chest x-rays that have an improved capability in detecting lung cancer. On one hand, chest x-rays have the advantage to be readily available, less costly and subjecting the patients to less radiation. On the other hand chest x-rays may not be as sensitive as CT’s in detecting small cancers. The factors that may contribute to missing a lung cancer on a conventional chest x-ray are related to lesion characteristics and location, technical factors and reader’s perception and errors. Interpreter performance and level of expertise play an important role in the detection and diagnosis of lung cancer.

The role of CAD as applied to chest x-rays is to minimize the potential errors by the interpreter and to identify potential cancerous lesions that can be missed. By improving the accuracy and consistency of radiological diagnosis, CAD will assist the radiologist in detecting early disease that can be potentially missed, allowing for early intervention and management^16^. We propose to use one of these systems in a screening setting. This will be the first controlled trial of chest x-ray screening in which there is a placebo control group, and the first chest x-ray screening trial that uses CAD to improve our ability to detect subtle cancers.

3.0 **OBJECTIVES**

3.1 **PRIMARY OBJECTIVE**

The primary aim of the study is to report and compare the frequency of advanced-stage lung cancers at the end of the 2 ½ year study between screened and placebo groups.

3.2 **SECONDARY OBJECTIVES**

The secondary aims are as follows:

- Determine whether lung cancer screening with chest x-rays and computer aided detection (CAD) reduces the incidence of symptomatic advanced lung cancer compared to no screening in a high-risk population. The presence of advanced symptomatic lung cancer will be determined by the Outcomes Review Committee.
- Compare the disease-specific mortality between screening with chest x-ray and CAD versus no screening. This is the endpoint used in many RCTs of lung cancer screening; thus, we will be able to compare our study results to the results of these other trials.
- Compare the all-cause mortality rate between screening with chest x-ray and CAD versus no screening. This endpoint includes all deaths including those attributed directly and indirectly to screening.
- Compare the length of time between randomization and the incidence of symptomatic advanced lung cancer for screened subjects versus controls. This analysis will provide data about how screening might delay the development of advanced lung cancer.
- Measure the monetary cost relative to the quality of life between screening with chest x-ray and CAD versus no screening. The comparison of quality of life since randomization between the two study arms will reveal differences over time due to screening. Coupled with an assessment of monetary costs, cost-utility of screening will be measured. Note that the cost-utility analysis will be performed on a subset of the study participants.

4.0 **SELECTION OF PATIENTS**

A 12-month accrual phase is planned. However, if we do not meet our accrual goal within 12 months, we will continue to enroll until March 31, 2010 or until our accrual goal is met; whichever happens first. Participants enrolled after December 31, 2009 will receive one screening at baseline; no incidence screening is planned for these participants. We anticipate enrollment of 8,000 participants.

4.1 **RECRUITMENT**

Recruitment strategies and methods will vary across sites, depending upon specifics of subject’s demographics and resources. All advertisements will be reviewed and approved by the IRB before implementation.

4.2 **INCLUSION CRITERIA**

The **inclusion** criteria for the study are as follows:

1. Ages 40-75 years and at least one of the following criteria…

(a) a current or ex-smoker with at least a 10 pack years history

(b) has first degree family member (parent, sibling, or child) with a history

of lung cancer

(c) has a diagnosis of Chronic Obstructive Pulmonary Disease (COPD)

2. Subject is able to return to the CCHS for annual follow-up screening.

3. Subject is willing to sign a medical release form.

4.3 **EXCLUSION CRITERIA**

The **exclusion** criteria are as follows:

1. Within the last 6 weeks, subject has had:

(a.) A new cough or chronic cough that has gotten worse

(b.) Either new shortness of breath, or any worsening of shortness of

breath

(c.) A cough producing blood

(d.) Constant chest pain

(e.) Respiratory infection, pneumonia, or cold

(f.) Unintentional and unexplained weight loss greater than 5% of total

body weight

2. Subject’s current health condition requires oxygen.

3. Subject has a medical condition that would prevent the subject from

undergoing treatment for lung cancer (e.g. uncorrectable, untreatable heart

condition, cirrhosis of the liver, HIV or AIDS)).

4. Subject has been diagnosed with a malignancy within the last 5 years,

excluding non-melanoma skin cancer, carcinoma in situ of the cervix and

localized prostate cancer

5. Subject has received a chest x-ray or CT within the last 6 months.

6. Subject is participating in another cancer screening trial.

7. Subject has had a pneumonectomy.

8. Subject has had a Lobectomy or Segmentectomy of the lung

within the last 5 years.

9. Subject is participating in an investigational drug or device study, a cancer

screening study, or a cancer prevention study (other than a smoking cessation

program)

5.0 **REGISTRATION PROCEDURE**

People interested in the study will be asked to call a toll free number, 1-866-505-LUNG (5864). Trained phone interviewers will be available to take these calls, provide a general description of the study and its goals, answer questions about the study, complete the detailed exclusion/inclusion criteria questionnaire, and schedule a baseline visit for eligible subjects. Eligible subjects will be sent a copy of the informed consent document to read prior to their scheduled appointment.

Eligible subjects will be scheduled for a baseline visit at various accrual areas within Cleveland Clinic Regional Healthcare Centers.

6.0 **STUDY DESCRIPTION AND RANDOMIZATION**

At the baseline visit, subjects will be asked to sign the consent form. The baseline study forms (see table 1) will then be administered by the study trained research personnel and entered online. After the completion of the baseline study forms, subjects will be randomized using an online randomization program. The study trained research personnel will log into a website, secured by passwords, to randomize patients. Study subjects will be randomized with equal allocation to either A) free screening with a chest x-ray interpreted by chest radiologists using computer-aided detection (CAD) or B) placebo screening chest x-ray. The subject’s randomization assignment will be determined by the program and recorded into the database. The allocation assignment will not be displayed. Rather, a randomization number will be displayed. The study trained research personnel will print the randomization number and give it to the trained radiology personnel. In the chest x-ray rooms, there will be a paper list which maps the randomization numbers to the study arms (i.e. chest x-ray or placebo). The trained radiology personnel will locate the randomization number on the paper list and determine whether the subject is to receive a chest x-ray or placebo. In this way both the study subjects and study trained research personnel will be blinded to the subjects’ study arm allocation.

A 4-variable stratified randomization design will be applied based on the following stratification variables: site of enrollment (main campus, east, west, or south), age (<65 –versus- >65 years of age), gender, and symptoms status (no reported chronic cough or shortness of breath -versus- reported chronic cough or shortness of breath). A blocked stratified randomization scheme will be used. There will be 8 strata within each enrollment site: 2 ages x 2 genders x 2 symptom statuses (total of 32 strata). Within each stratum we will use a randomized block size of variable size for that stratum.

7.0 **TECHNICAL DESCRIPTION OF CHEST X-RAY**

Patients selected to undergo a chest x-ray will be subjected to the standard procedure in which a frontal view would be obtained. The test will be performed at different locations using digital equipment that is available throughout the Cleveland Clinic Health System (CCHS).

The standard technical factors will be:

PA View

125kVp

1.60mAs

500ms

Left and right AEC sensors

72” SID

The radiation dose to the patient is 20 millirems for the frontal view (PA). That is less than a 6% of the yearly radiation dose that every person is exposed from normal background radiation (360 millirems). It should also be noted that the radiation generated by the chest x-ray procedure is limited to the chest organs while the background radiation affects the whole body.

Patients that will be selected to be in the control group will be introduced to a chest x-ray room and be placed against the x-ray unit however no exposure will be triggered and no image will be obtained. There will be no radiation exposure to the patient.

8.0 **INTERPRETATION OF CHEST X-RAY AND CAD**

Chest x-rays will be interpreted by chest-radiologists. The radiologist will first read the case without CAD, and then with CAD. The findings will be reported by the radiologist on the Radiology Reading Form (case report form), which includes reader findings and recommendations for follow-up. If the radiologist does not find an actionable nodule and does not recommend follow-up for the subject, then the images will be read by a second chest radiologist. This second reading will be blinded to the first interpretation. The findings will be reported by the second radiologist on Radiology Reader Form (case report form). Subjects who underwent a chest x-ray and were found to have an actionable finding on the chest x-ray by one of the radiologists will be mailed a Findings Report form, along with the dictated medical record reports, via certified mail. The Findings Report form will include all actionable findings found by the radiologist. Chest x-rays will be interpreted and reported within approximately four to six weeks of the chest x-ray, and subjects with actionable findings will be notified shortly thereafter.

Subjects with actionable findings will be phoned after written notification to answer any questions the subject has, and to encourage a follow-up visit with a primary care physician or pulmonologist. The recommendations of the radiologist will not be mandated to the subject’s physician; rather, subjects and their physicians together will determine the plan for follow-up testing and treatment, as appropriate.

Subjects with no primary care physician will be offered a list of physicians who could receive the results of their positive screening and oversee the management of follow-up recommended as a result of screening. Under-insured participants will be offered information about local sources of financial assistance and health care services. We will limit the number of subjects that are uninsured at baseline to 10% to ensure that the study objectives are not compromised by an inadequate number of subjects who pursue recommended follow-up.

9.0 **ANNUAL SCREENINGS**

Subjects without a diagnosis of lung cancer and who are deemed to be able to undergo treatment for lung cancer if detected (i.e. no medical condition that would prevent the subject from undergoing treatment for lung cancer) will be scheduled for an annual screening (or placebo screening). A study trained research personnel will phone the study subject and set up an appointment. One incident screens/placebo screen per subject is planned.

At this annual visit, the study trained research personnel will administer the annual study forms (see table 1) to the subject and record the data online. The study trained research personnel will then give the radiologist technician the subject’s randomization number. The trained radiology personnel will locate the randomization number on the paper list in the chest x-ray rooms and determine whether the subject is to receive a chest x-ray or placebo.

The chest x-rays from the annual incident screen will be interpreted by chest radiologists. A radiologist will first read the case without CAD, and then with CAD. Subjects who underwent a chest x-ray and were found to have an actionable finding on the chest x-ray will be mailed a Findings Report form, along with the dictated medical record reports, via certified mail. The Findings Report form will include all actionable findings found by the radiologist. If the radiologists does not recommend follow-up for the subject; then the images will be read by a second chest radiologist. This second reading will be blinded to the first interpretation. The findings will be reported by the second radiologist on a Radiology Case Report Form. Chest x-rays will be interpreted and reported within approximately four to six weeks of the chest x-ray, and subjects with actionable findings will be notified shortly thereafter. Subjects and their physicians together will determine the plan for follow-up testing and treatment, as appropriate.

10.0 **SUBJECT FOLLOW-UP**

At the time of study enrollment and at the annual screen, subjects will be given a packet of study forms (see table 1) and a pre-addressed envelope to take home. The study forms are to be completed by the subject at home and returned to the study trained research personnel at six-month intervals. If the study subject does not return the forms within 2 weeks of the scheduled completion date, the study trained research personnel will phone the subject and administer the study questionnaires via telephone. The study trained research personnel will review the study forms for completeness. If the study trained research personnel have questions about the study form, then the study trained research personnel will call the subject for clarification before sending the study form for data entry into the database.

If at anytime during the duration of the study, the subject is diagnosed with lung cancer additional follow-up will be obtained regarding the diagnosis, pathology staging, treatment, complications and mortality at 2 months intervals. This information will be obtained via telephone or mail by the study trained research personel.

11.0 **RECORD KEEPING**

Records will be secured and kept per all institutional, NCI, state and Federal regulations.

11.1 **DATA COLLECTION FORMS**

The forms to be completed by the study subjects are attached as appendices to this protocol.

Potential trial participants will call into the trial hotline number. Trained research personnel will obtain information to screen for possible eligibility and collect contact information for study appointment. The investigator complies and agrees the protected health Information (PHI) collected to identify eligibility of candidates adheres to the following three criteria: is necessary for the purpose of this research, will be used solely for this research, and will not leave The Cleveland Clinic Health System (CCHS).

The authorization for the release of medical information form will be signed by all subjects at the time of randomization. Attempts to obtain the authorization will be made every six months, but will be required yearly. We will be able to capture changes to their medical insurance provider on a yearly basis.

The eligibility form will be completed to confirm that the subject meets inclusion and exclusion criteria prior to enrolling subject into research trial. Patient informed consent will be signed prior to randomization.

The baseline form collects data on age, race, gender, education level, work status, income range, previous and existing medical conditions, family history of medical conditions, and occupational exposures. It will be administered to all subjects by the study trained research personnel and entered online at study entry. This information will allow us to characterize our study population.

Every six months all subjects will complete a follow-up form and a medical utilization form to capture data on physician visits in the previous 6 months. If any study subject reports that he has been diagnosed with lung cancer, then we will contact the institution they cited on their medical release form and request their medical records. In addition, if a CCHS patient reports medical visits within the last 6 months that are suspicious for lung cancer or of lung cancer diagnosis, we will access their EPIC records if the visit took place at the CCHS; if the visit took place outside of the CCHS, we will contact the institution where the visit took place and request their medical record. If the medical records cannot be achieved for any special reasons, the cost for such visits will be imputed, based on other similar visit types.

The DRGs (Diagnosis Related Groups) coded for hospitalizations will be used to assign standard Medicare reimbursement costs based on the MedDRA code. For non-hospitalizations, standard Medicare costs will be derived from the Physician Fee schedule using relevant CPTs (Current Procedural Terminologies) reported by the billing records of patient’s healthcare institution, when possible, or appropriate CPTs assigned by the study team for healthcare utilization by sites who cannot provide the CPTs for study patients. Missed work and additional help with healthcare and housework due to the disease from patients, their families, and caregivers will be captured on the follow-up forms to calculate the indirect costs of the subjects from the societal perspective.

The radiology Form will be completed by the chest radiologist at baseline and year one. The following information will be collected; the number of actionable nodules, location, size, contour, shape, non-calcified density, notable findings.

The UCSD Shortness of Breath Questionnaire rates the breathlessness experienced by the subject when he/she does or were to do certain tasks. This questionnaire will be completed by all study subjects every six months.

The St. George Respiratory questionnaire and the UCSD shortness of breath questionnaire capture data on the most common symptoms of lung cancer. The questionnaires address the frequency and severity of symptoms, along with how these symptoms affect everyday activities. These questionnaires will be completed by all study subjects every six months.

The EQ-5D (Euro-Qol – 5 dimension) is a standardized, validated, internally consistent quality of life questionnaire specifically designed for measuring health related quality of life. It provides a simple descriptive profile and a single preference-based index for global health status. The EQ-5D was originally designed to complement other instruments such as the SF-36 or disease-specific questionnaires but is now increasingly used as a 'stand alone' measure. The EQ-5D has recently been weighted according to the social preferences of the US population by Shaw et al (2005). The corresponding set of EQ-5D health state preferences will be used to derive the global health related quality of life for our study. There are in excess of 800 published references to the EQ-5D and this measure will be used more frequently for US studies now that the US population preference norms are available. This questionnaire will be completed by all study subjects every six months.

The Paper Standard Gamble questionnaire^17^ will be used to collect patients’ utility outcome (effectiveness) of the cost-effectiveness analysis. With Standard Gamble, we are asking how much risk the patient is willing to take for a chance of perfect health. Because it involves uncertainty, while EQ-5D does not, Standard Gamble is the only true utility measure. Originally, a facilitator used props during a face-to-face encounter with the patient. This method is expensive because it requires the facilitator’s time. For ease-of-administration reasons, the paper-base tool (Paper Standard Gamble) was designed and good results have been achieved.^18^ This form will be administered by trained research personnel at baseline and yearly thereafter for the subset of study subjects who are CCHS patients.

Table 1: Study Forms

| **Study Form** | **Screening** | **Baseline** | **Every 6 Months**  +/- 2 weeks | **Annual**  **Visits**  +/- 1month | **Malignant Nodule**  Additional follow-up  Every 2 Months |
| --- | --- | --- | --- | --- | --- |
| Screening Form | X |  |  |  |  |
| Eligibility Form |  | X |  |  |  |
| Medical Release Form |  | X | X | X | X  (Obtain as needed) |
| Baseline Visit Form |  | X |  |  |  |
| Follow-up / Medical Utilization Form |  |  | X | X | X |
| Radiology Reading Form  (with and with out CAD) |  | X |  | X |  |
| Nodule Form |  | X | X | X | X |
| Shortness of Breath Questionnaire (UCSD) |  | X | X | X |  |
| St. George’s Respiratory Questionnaire |  | X | X | X |  |
| EQ-5D Questionnaire |  | X | X | X |  |
| Paper Standard Gamble Questionnaire* |  | X |  | X |  |

*Administered to CC patients only; the cost effectiveness analysis will be performed only on subjects who are CC patients at the time of study enrollment.

11.2 **MISSING AND DELINQUENT DATA**

The study trained research personnel will review incoming paper forms for clarity and completeness of the relevant information. If there are questions or missing data the study trained research personnel will telephone the patient for clarification.

11.3 **DATABASE**

The CC’s department of Quantitative Health Sciences will serve as the Data Coordinating Center (DCC) for this study. Data management will be hybrid in nature, in that data will be transmitted to the DCC from a variety of sources, to reside in a central database. In addition, a small amount of data will originate at the DCC, i.e. randomization information.

The basic data management principles include: 1. The DCC will hold the central database. All data originate from validated data systems, transferred to the CC DCC. All electronic data capture or upload processes will be completely tested and validated, but represent a small part of the entire process, in that, data dictionaries and variable definitions are predefined by the originating source.

There are four data sources for this study: 1. The demographics, EQ-5D and paper standard gamble, symptoms report, and medical resource utilization forms, completed by study subjects; 2. radiology report forms completed by individual radiologists; 3. randomization information maintained by the DCC; and 4. Medicare DRG and CPT information (costs related). With the exception of the randomization information, all data originate outside of the DCC. Data are entered through a validated web-enabled user interface, and electronically captured and stored at the DCC

A web-enabled application utilizing system and user based permissions will allow the clinical sites to enroll, randomize, and collect study related information within the DCC centralized database. The application will utilize data field validation when appropriate to ensure data entry/capture accuracy of key variables. The central data repository is an Oracle database stored on state-of-the-art servers with the DCC’s physically secure data center. The DCC utilizes industry best practices to ensure data quality, data security and data backup.

At regular intervals, data will be queried by the DCC using Oracle Discoverer and Oracle Reports to perform consistency checks on key variables and between forms. As required, data will be extracted from the master Oracle database to SAS datasets for statistical analysis. Each dataset will contain a data dictionary, a complete description of how the data was extracted and version identification as well as key dates and timing sequences.

11.4 **RANDOMIZATION PROGRAM**

A restricted randomization will be conducted centrally by the Cleveland Clinic (CC), Data Collection Center(CC/0 in a manner designed 1) to limit the probability of substantial imbalance in the allocation of subjects to study arms within a site, and 2) to limit the probability of substantial imbalances between study arms in the prognostic stratification variables.

Randomization will be implemented using a secure web-enabled system residing at the CC. A unique subject identification number and site location will be assigned to each subject. Randomization will be implemented by informing the enrolling site, of the subject assignment to screening or placebo screening. A subject will be considered to have been randomized to the assigned study arm and included in the intent-to-treat population once the subject identification number has been assigned.

12.0 **END POINT ASSESSMENT**

12.1 **PRIMARY ENDPOINT ASSESSMENT**

The primary outcome measure is the frequency of advanced-stage cancers detected during the course of the 2 ½ year study. These advanced-stage cancers may be detected during the study's planned screenings, through progression of preclinical disease to clinical symptomatic disease, and, less frequently, during medical testing/screening for other purposes (e.g. presurgical work-up). Advanced-stage cancers will be defined as stage III or IV cancers and will be assessed as clinically indicated, which usually includes PET imaging, additional invasive testing (i.e. tissue sampling), and sometimes brain imaging.

12.2 **SECONDARY ENDPOINT ASSESSMENT**

A secondary endpoint is the presence/absence of symptomatic lung cancer. To assess this outcome, an expert outcome review committee, consisting of a pulmonologist and an oncologist, will be convened at the end of the study (after March 2011). The committee members, each working independently, will review all cases of diagnosed, pathology-proven lung cancer as well as all deaths. Committee members will be blinded to the randomization assignment of the subjects and results of screening tests. Based on the subject’s self-reported symptoms assessment during the study and their medical records, and death certificates (if appropriate and available) the committee members will determine whether the subject is asymptomatic or has symptoms that correlate with their diagnosis of lung cancer. Subjects determined to have symptoms of lung cancer by both committee members will be classified as events for calculation of this secondary endpoint. In cases where the two committee members disagree, the committee members will meet to discuss the cases and reach consensus. Deaths due to lung cancer and deaths due to the management or treatment of diagnosed lung cancer will also be classified as events. For all events, committee members will decide on, to within 6 months, the time at which the subject developed symptoms.

Note that for the assessment of the primary and secondary endpoints, subjects who are determined to have lung cancer that has metastasized will be considered a censored event at the time of diagnosis. These subjects’ lung cancer will not be classified as events in the assessment of the primary and secondary endpoints.

Definition of "Advanced Symptomatic Disease":

The following criteria will be assessed by the Outcomes Review. All three criteria must hold.

1. The patient has been diagnosed with non-small cell carcinoma, stage II or

higher, or small cell carcinoma, any stage.

2. The patient has experienced a symptom that led them to seek contact with a

physician since the time of the last study visit; or at the time of their next study

visit, the patient has developed a new symptom or a change in a chronic

symptom within the past 6 weeks, that did not yet lead to a doctor's visit.

3. The symptom(s) described in #2 are felt to be related to the diagnosis of lung

cancer as adjudicated by the Outcomes Review Committee.

For the other secondary endpoints, the committee will review the medical records and self-reported symptoms assessment of all deaths and determine if the death is due to lung cancer.

13.0 **STATISTICAL ANALYSIS PLAN**

13.1 **PRIMARY AIMS:**

Analyses will be based on an intent-to-treat perspective. We will test the null hypothesis that at the time of diagnosis the frequency of advanced-stage cancers is the same for screened and unscreened groups; the alternative hypothesis is that the frequency of advanced-stage cancers is different for screened and unscreened groups. These hypotheses will be tested at the end of the two and a half-year study, i.e. after March 31, 2011. The frequency of advanced-stage cancers in the two study arms will be compared using logistic regression analysis. A model will be fit to describe the probability of advanced-stage cancer as a function of the following independent variables: age, gender, symptoms status at the time of enrollment into the study (i.e. symptoms present or not present), study arm, and two-way interactions with study arm. The fit of the model will be assessed using the Hosmer-Lemeshaw goodness of fit test. The coefficients for study arm and interactions with study arm will be assessed, after adjusting for other covariates, using a likelihood ratio test with significance level of 0.05 (two-tailed).

13.2 **SECONDARY AIMS:**

One of the secondary aims of the study is to determine whether lung cancer screening using chest x-rays and CAD reduces the incidence of symptomatic advanced lung cancer relative to no screening. The observed cumulative incidence of symptomatic advanced lung cancer, measured in events per person per years observed, will be compared between the two study arms. The analysis will be carried out from an intent-to-treat perspective.

The incidence rate of symptomatic lung cancer will be estimated as follows

Incidence rate at time *t*= [# events by *t*] / [# of person-years at risk until *t*].

where “event” is the development of symptoms due to lung cancer or death due to treatment for the disease or lung cancer death (whichever is observed first). These events will be determined by the outcome review committee at the end of the study (after March 2011). The number of person-years at risk is equal to t′ – e, where t′ is the smaller of [time at which the subject experienced symptoms of lung cancer, time at which the subject was lost to follow-up if the subject did not die from lung cancer or from its treatment before *t*, time at which the subject died from lung cancer or its treatment, *t*], and

e is the number of study months before the subject was enrolled into the study (i.e. e=0 at the start of the study).

The following null hypothesis will be tested: the incidence rates of the screened and control subjects are equal. The alternative hypothesis is the incidence rates of the screened and control subjects are not equal. A stratified analysis will be used. There will be four strata:

- age < 65 years at enrollment; none/mild pulmonary symptoms at enrollment
- age > 65 years at enrollment; none/mild pulmonary symptoms at enrollment
- age < 65 years at enrollment; moderate pulmonary symptoms at enrollment
- age > 65 years at enrollment; moderate pulmonary symptoms at enrollment.

Note that only four strata were chosen because the overall number of events will be small. These particular four strata were chosen because we expect that age and pulmonary health at enrollment are the strongest predictors of the development of lung cancer and curability of lung cancer.

For each stratum i, the data can be summarized as follows:

Screened Control Total

# events a_1i_ a_0i_ M_i_

Person-years N_1i_ N_0i_ T_i_

______________________________________________________________________________

The Mantel-Haenszel rate ratio^19^ is given by

RR=[∑a_1i_N_0i_/T_i_] / [∑a_0i_N_1i_/T_i_].

The variance of the ln(RR) is given by^20^

Var[ln(RR)] = [∑M_i_N_1i_N_0i_/T_i_^2^] / [(∑a_1i_N_0i_/T_i_)(∑a_0i_N_1i_/T_i_)].

The 95% CI for RR is as follows:

lower = exp[ln(RR) – zval * Var(ln(RR))^1/2^

upper = exp[ln(RR) + zval * Var(ln(RR))^1/2^,

where zval is the value from the standard normal distribution whose value will depend on the time of the analysis (see subsection on Interim Analyses).

If the 95% CI for RR does not include the value one, then we will reject the null hypothesis.

The analyses described above for the cumulative incidence of symptomatic advanced disease will also be performed for disease-specific mortality and all-cause mortality.

A Cox proportional hazards model will be fit to compare the two study arms on the time from randomization until symptomatic disease. Covariates will be included in the model, such as age, gender, race, smoking history, symptoms at presentation, as well as site of enrollment. A significance level of 0.05 will be applied.

13.3 **OTHER ANALYSES:**

In the intervention arm, for each screening, we will report the number of subjects with actionable findings and the type of findings. We will report the follow-up results of these findings. This will provide data on the false positive rate and the size and stage of detected cancerous nodules.

We will test the effect of CAD on radiologist performance. This will include the effect of CAD on the radiologists’ sensitivity, as well as the effect on the false positive rate. First, we will separate the screened cases into one of three groups based on follow-up results, as available: cancerous nodule(s) present, benign nodule(s) present and no cancerous nodules present, no nodules present. For each group we will compare the frequency with which radiologists reported an actionable nodule without CAD versus with CAD. For each group the frequencies will be compared using a McNemar’s test, modified for clustered data.^21^  The modification is necessary because two radiologists will be interpreting each case. A significance level of 0.05 will be applied.

14.0 **SAMPLE SIZE CONSIDERATIONS**

The following assumptions were made for determining the required sample size for the primary study aim: prevalence of cancer at baseline screen=1%; annual incidence of cancer=0.5%; annual lost to follow-up=10%; 90% of people randomized to screening are compliant at each screening; 12-month recruitment period; 1 incident screen planned for 12 months after the initial prevalence screen; and 85% sensitivity of chest x-ray with CAD. For development of symptoms following the development of preclinical disease, we assume a uniform distribution over 30 months so that the average time is 16 months from development of detectable preclinical disease until development of symptoms. We assume a 70% reduction in the development of advanced-stage cancers if preclinical disease is detected at screening.

*Placebo Arm:*

Based on these assumptions, if N patients are randomized to the placebo arm, then after one year the number of symptomatic, advanced-stage cancers will be:

(1-lost-to-follow-up rate)(prevalence) (rate of development of symptoms over 12 months)(N).

Similarly, at year two the number of symptomatic, advanced-stage cancers will be approximately:

(1-lost-to-follow-up rate)^2^ (prevalence) (rate of development of symptoms over 24 months)(N)

for those with preclinical disease at the time of enrollment.

In addition, there are

(1-lost-to-follow-up rate)(incidence)(rate of development of symptoms over 12 months)(N’)

symptomatic, advanced-stage cancers from those who develop lung cancer within the first year, where N’=N-(N)(prevalence).

*Screened Arm:*

If N patients are randomized to the screening arm, and if we assume that at the time of baseline screening, 30% of detected preclinical disease is advanced-stage cancer, then the number of detected advanced-stage cancers detected at baseline is M, where

M = (prevalence)(sensitivity) (0.30)(N).

After one year the number of advanced-stage cancers will be approximately:

(1-lost-to-follow-up rate) (prevalence)(sensitivity)(rate of development of symptoms over 12 months) (N-M)(1-risk reduction due to early treatment) for those cancers detected at baseline but still progressed to symptomatic, advanced-stage cancers in one year.

In addition, there are those cancers undetected at baseline that progress to symptomatic, advanced-stage cancers in the first year:

(1-lost-to-follow-up rate)(prevalence)(1-sensitivity) (rate of development of symptoms over 12 months)(N-M).

At the one-year incident screen we assume that 15% of detected preclinical disease is advanced-stage cancers. Thus, at the incident screen, M’ advanced-stage cancers will be detected:

M’= (1-lost-to-follow-up rate)(compliance rate with screening)(incidence rate)(sensitivity) (0.15)(N’).

At year two, the number of symptomatic, advanced-stage cancers will be approximately:

(1-lost-to-follow-up rate)^2^ (prevalence)(sensitivity) (rate of development of symptoms over 24 months)(N-M)(1-risk reduction due to early treatment) for those with preclinical disease detected at the time of enrollment but still progressed to advanced-stage cancers by two years.

In addition, there are those cancers undetected at baseline that progress to symptomatic, advanced-stage cancers over 24 months: (1-lost-to-follow-up rate)^2^ (prevalence)(1-sensitivity) (rate of development of symptoms over 24 months)(N-M).

There are also cancers that developed over the first year, were detected at the incident screen, and became symptomatic, advanced-stage cancers during the second year:

(1-lost-to-follow-up rate)^2^ (compliance rate with screening)(incidence)(sensitivity)(rate of development of symptoms over 12 months)(N’-M’)(1-risk reduction due to early treatment).

Finally, there are cancers that developed over the first year, were undetected at the incident screen, and became symptomatic, advanced-stage cancers during the second year:

(1-lost-to-follow-up rate)^2^ (incidence)(1-sensitivity)(rate of development of symptoms over 12 months)(N’-M’).

Table 2 summarizes the number of advanced-stage lung cancers expected in the two study arms as a function of N.

| N, # subjects per study arm | # advanced-stage cancers in placebo arm | # advanced-stage cancers in screening arm | Total Number of advanced-stage cancers |
| --- | --- | --- | --- |
| 3000 | 35.6 | 23.2 | 58.8 |
| 3500 | 41.6 | 27.1 | 68.7 |
| 4000 | 47.5 | 30.9 | 78.4 |
| 4500 | 53.4 | 34.7 | 88.1 |
| 5000 | 59.3 | 38.7 | 98.0 |

We plan to fit a logistic regression model to describe the probability of advanced-stage lung cancer as a function of 1) patient age, 2) gender, 3) symptoms status at time of enrollment, and 4) study arm randomized to. We will also consider in the model all two-way interactions with study arm. The rule of thumb to avoid model overfitting is at least 10 events per independent variable in the model, thus we need at least 70 advanced-stage cancers (total) for the model building.

We estimate that if the frequency of advanced-stage cancers at two years is 1.19% for controls and 0.77% for the screened arm (difference of 0.42%), then we can detect this difference with 4000 subjects per study arm with 77% power (5% type I error rate, two-tailed test).

15.0 **COST-EFFECTIVENESS ANALYSIS**

The cost effectiveness analysis will be performed only on subjects who declare themselves as Cleveland Clinic patients at entry into the study. The rationale is based on experiences from other studies where it was found to be impossible to collect valid medical visit and hospital stay data from other medical institutions even when the study subject had signed a medical release form. Thus, it was decided that the cost-effectiveness assessment in this study would be limited to Cleveland Clinic patients for whom medical records could more easily and reliably be obtained. We recognize that this decision limits the generalize ability of our cost effectiveness analysis results.

This analysis will be conducted from the US societal perspective as recommended by the U.S. panel for cost effectiveness in health and medicine.^24^ The initial time horizon will be the three-year study period, but will also be extended to 10 and 20 years using Markov modeling approach to measure the long-term cost-effectiveness difference between lung cancer screening and placebo screening. When data are collected after each visit, the intermediate analysis will be conducted to analyze the short-term cost-effectiveness outcomes if necessary. The results from the intermediate study could be used to guide the concurrent treatment in clinical settings.

Two categories of data are collected for the cost-effectiveness analysis: cost data and effectiveness data. The cost data will include both direct cost and indirect cost for patients with screening or placebo screening. The direct cost will include costs of inpatient care, emergency-room visits, outpatient care, physician visits, and laboratory tests. The data will be collected by medical utilizations based on case report from (CRF). The cost of lung cancer screening by CAD chest x-rays will be included for patients in the screening group too. Current procedural terminology (CPT) and diagnosis related group (DRG) codes will be collected from the billing system for Cleveland Clinic internal visits. Codes for external visits will be assigned by healthcare institutions that treat our patients and can provide these codes to us. Otherwise, CPT and DRG codes will be determined by the project team based on the data collected on the medical utilization forms provided by the patient or their healthcare institutions. If a patient is hospitalized after randomization, the primary reason and length of stay will be collected too. This will be converted to a DRG code, which will be used to derive costs based on Medicare reimbursement amount. In addition, use of oxygen and the amount of oxygen use will be collected as prescription information, and the cost of oxygen will be calculated based on the standardized average wholesale price (AWP) reported in the Drug Topics® Red Book^25^. There is no reason to believe that the use of other prescription medications will differ significantly between the screening and placebo screening group. Thus the incremental cost is assumed zero and no additional prescription information will be collected.

The indirect cost comes from the loss of work or loss of productivity due to the screening and the diseases. From the society perspective, such costs associated with both patients and their family members or caregivers who take care of the patients will be collected. During each six-month visit, loss of work-days (hours) from the last visit to this visit will be collected including the time of hospitalizations, clinical visits, traveling to/from the clinic. The loss of productivity and time-related costs will be calculated as a product of days (hours) lost by their salary range determined at baseline. The indirect cost of caregivers will be collected in the same manner by multiplying the average salary and time spent from the caregivers. All cost data will be inflated to a common year using the medical care inflation indices from the Bureau of Labor Statistics (http//www.bls.gov).

A cost-effectiveness model developed in Treeage Data^®26^ pro suite will be utilized to investigate the cost effectiveness of lung cancer screening with chest x-rays and CAD in asymptomatic patients compared to the similarly profiled cohort of patients who receive placebo screening. The cost of all healthcare utilizations as calculated above will be included in the model. The health related quality of life (utility) results of the EQ-5D and paper standard gamble will be used as the effectiveness data so that results may be expressed as cost per quality adjusted life years (QALYs).^27^ Discount rates of 3% will be used for both costs and outcomes in this cost-effectiveness model, as recommended by the U.S. panel for cost effectiveness in health and medicine.^24^

The incremental cost effectiveness ratio (ICERs) will be calculated for the base analysis. Bootstrap simulation with replacement will be utilized to derive mean costs and utilities and associated ICERs with 95% confidence intervals.^28^ Acceptability Curves will be constructed to determine the probability of lung cancer screening being cost effective under certain cost-effectiveness thresholds.^29^ Conventionally, ICERs of less than $50,000 per QALY are considered very cost effective whilst those with an ICER of between $50,000 and $100,000 per QALY are considered moderately cost effective.^30^

The Markov decision tree model will be used to derive the long-term cost-effectiveness differences between lung cancer screening and placebo screening for 10 and 20 years. The transition cycle of the Markov model will be one year. The transitional probabilities between different disease states will be calculated from the collected incidence of the study. The cost and utility data of the Markov model will apply the data from the five-year study period by discounting of 3% as a base-case. Similarly, ICERs will be calculated and bootstrap simulation will be used to derive the 95% confidence intervals of the ICER.

Sensitivity analysis will be performed by varying costs and utilities by their 95% confidence intervals established in our study. The impact of varying the probabilities of the detection of pre-clinical disease and incidental findings will also be investigated in sensitivity analysis. Sub-group analysis will also be used to investigate the patient characteristics that could contribute to the most cost-effective use of screening, including age, gender, and smoking history.

16.0 **ADVERSE EVENTS**

The principal investigator/s will review all adverse events and unanticipated problems as related to the imaging research. Adverse events and unanticipated problems will be reported promptly per all institutional, FDA, state and Federal regulations. In addition, if the investigator and co-investigators as a group have any concerns, they will escalate those concerns to the outside advisory board for review and guidance.

Adverse event reporting is to be distinguished from the collection of data for purposes of analyzing trial endpoints, which is achieved through the recording of specific data elements on case report forms and statistical analysis.

Complications associated with primary interventions are termed direct AE's. Screening tests promote downstream diagnostic interventions: complications associated with these diagnostic interventions are termed indirect AE's. Primary interventions in this protocol are the screening chest x-ray or placebo. In this protocol only direct adverse events associated with the primary trial intervention (chest x-ray or placebo) will be reported as adverse events. Indirect adverse events (complications associated with these diagnostic interventions) will be documented as part of the endpoints on case report forms.

Potential expected and unexpected adverse events may include but are not limited to any injury or incident occurring to the patient while receiving the primary trial intervention or possibly accidental radiation exposure when a placebo was originally intended

For this protocol, the following AE's are specifically excluded from AE reporting unless they are related to the primary trial intervention (chest x-ray or placebo): Complications of the following conditions: hospitalizations, prolonged hospitalization, or surgeries should NOT be reported as an AE in this trial.

Complications from diagnostic procedures performed because of screening intervention: such as elective surgery or minimally invasive procedures for a pre-existing condition, therapy for lung cancer, death from lung cancer, death from other cancer or pre-existing condition. These conditions will be recorded on study report forms for the purpose of endpoint analysis.

17.0 **DATA SAFETY AND MONITORING COMMITTEE**

These members will meet at least once per study year by conference call and/or in person to review adverse events and interim analyses, and advise the research team.

18.0 **PATIENT CONSENT AND PEER JUDGEMENT**

All institutional, NCI, FDA, state and Federal regulations concerning informed consent and peer judgment will be fulfilled.

19.0 **REFERENCES**

1. Jemal A, Siegal R, Ward E, et al. Cancer statistics, 2006. CA Cancer J Clin. 2006;56:106-130.
2. Community Health Assessment and Center for Public Health Data and Statistics. 2002, Ohio Department of Health Columbus OH.
3. Annual Smoking Attributable Mortality; Years of Potential Life Lost and Economic Cost – United State – 1995/199, Morbidity and Mortality weekly report, MMWR, 2002. 51; pp.300-303.
4. Manser RL, Irving LB, Byrnes G, et al. Screening for lung cancer: a systematic review and meta-analysis of controlled trials. Thorax 2003;58:784-789.
5. Henschke CI, McCauley DI, Yankelevitz DF, et al. Early Lung Cancer Action Project: overall design and findings from baseline screening. Lancet. 1999;354:99-105.
6. Henschke CI, Naidich DP, Yankelevitz DF, et al. Early Lung Cancer Action Project: initial findings on repeat screening. Cancer. 2001;92:153-159.
7. Nawa T, Nakagawa T, Kusano S, et al. Lung cancer screening using low-dose spiral CT. Results of baseline and 1-year follow-up studies. Chest. 2002;122:15-20.
8. Swensen SJ, Jett JR, Sloan JA, et al. Screening for lung cancer with low-dose spiral computed tomography. Am J Respir Crit Care Med 2002;165:508-513.
9. Swensen SJ, Jett JR, Hartman TE, et al. Lung cancer screening with CT: Mayo Clinic experience. Radiology 2003;226:756-761.
10. Swensen SJ, Jett JR, Hartman TE, et al. CT screening for lung cancer: Five-year prospective experience. Radiology 2005:235:259-265.
11. Pastorino U, Bellomi M, Landoni C, et al. Early lung-cancer detection with spiral CT and positron emission tomography in heavy smokers: 2-year results. Lancet 2003;362:593-597.
12. Mahadevia PJ, Fleisher LA, Frick KD, et al. Lung cancer screening with helical computed tomography in older adult smokers. A decision and cost-effectiveness analysis. JAMA. 2003;289:313-322.
13. The International Early Lung Cancer Action Program Investigators. Survival of patients with stage I lung cancer detected on CT screening. N Engl J Med 2006;355:1763-1771.
14. Smith RA, von Eschenbach AS, Wender R, etal. American Cancer Society guidelines for the early detection of cancer; update of early detection guidelines for prostate, colorectal, and endometrial cancers. Also: Update 2001-testing for early lung cancer detection. CA Cancer J Clin. 2001;51:38-75.
15. Bach PB, Niewoehner DE, Black WC. Screening for lung cancer. The guidelines. Chest. 2003;123:83S-88S.
16. Kakeda S, Moriya J, Sato H, et al. Improved detection of lung nodules on chest radiographs using a commercial computer-aided diagnosis system. AJR 2004; 182:505-510.
17. Kattan MW. Comparing treatment outcomes using utility assessment for health-related quality of life. Oncology 2003;17:1687-1693
18. Ross PL, Littenberg B, Fearn P, et al. Paper standard gamble: A paper-based measure of standard gamble utility for current health. Int J Technol Assess Health Care 2003;19:135-147
19. Rothman KJ, Boice JD. Epidemiologic analysis with a programmable calculator. Brookline, MA. Epidemiology Resources, 1982.
20. Greenland S, Robins JM. Estimation of a common effect parameter from sparse follow-up data. Biometrics 1985; 41: 55-68.
21. Obuchowski, NA. On the Comparison of Correlated Proportions for Clustered Data. Statistics in Medicine, 1998; 17: 1495-1507.

22. Lan KKG, DeMets DL. Discrete sequential boundaries for clinical trials. Biometrika 1983;

70:659-663.

23. O’Brien PC and Fleming TR. A multiple testing procedure for clinical trials. Biometrics

1979; 35: 549-556.

24. Gold MR, Siegel JE, Russell LB, Weinstein MC. Cost Effectiveness in Health and Medicine.

In: Press OU, ed. New York, 1996.

25. Pharmacy's Fundamental Reference, 2006 edition. Montvale, NJ:

Thomson PDR, Red Book; May 2006.

26. TreeAge Pro 2006 Suite. [http://www.treeage.com](http://www.treeage.com/). Williamstown, MA, 2006

27. Meunnig P. Designing and Conducting Cost-Effectiveness Analyses in Medicine and Health

Care. San Francisco, CA: Jossey-Bass; 2002.

28. Briggs AH, Wonderling D, Mooney C. Pulling Cost-Effectiveness Analysis up by its

Bootstraps: A non-parametric Approach to Confidence Interval Estimation. Econometrics

and Health Economics 1997; 6:327 - 340.

29. O'Brien BJ, Briggs AH. Analysis of uncertainty in health care cost-effectiveness studies: an

introduction to statistical issues and methods. Stat Methods Med Res 2002; 11:455-68.

30. Tengs T and Wallace A. One thousand health related quality of life estimates. Medical Care

2000;38: 583 – 637.

20.0 **APPENDIX**

A. Finding Letters

B.Case Report Forms

C. Advertisement
